# Measuring and Modeling Cue Dependent Spatial Release from Masking in the Presence of Typical Delays in the Treatment of Hearing Loss

**DOI:** 10.1177/23312165221094202

**Published:** 2022-04-27

**Authors:** Julian Angermeier, Werner Hemmert, Stefan Zirn

**Affiliations:** 1 Peter Osypka Institute of Medical Engineering, Faculty of Electrical Engineering, Medical Engineering and Computer Sciences, 64369 University of Applied Sciences Offenburg; 2 Bio-Inspired Information Processing, Munich Institute of Biomedical Engineering, 9184 Technical University of Munich

**Keywords:** binaural hearing, spatial release from masking, speech in noise, auditory model

## Abstract

In asymmetric treatment of hearing loss, processing latencies of the modalities typically differ. This often alters the reference interaural time difference (ITD) (i.e., the ITD at 0° azimuth) by several milliseconds. Such changes in reference ITD have shown to influence sound source localization in bimodal listeners provided with a hearing aid (HA) in one and a cochlear implant (CI) in the contralateral ear. In this study, the effect of changes in reference ITD on speech understanding, especially spatial release from masking (SRM) in normal-hearing subjects was explored. Speech reception thresholds (SRT) were measured in ten normal-hearing subjects for reference ITDs of 0, 1.75, 3.5, 5.25 and 7 ms with spatially collocated (S_0_N_0_) and spatially separated (S_0_N_90_) sound sources. Further, the cues for separation of target and masker were manipulated to measure the effect of a reference ITD on unmasking by A) ITDs and interaural level differences (ILDs), B) ITDs only and C) ILDs only. A blind equalization-cancellation (EC) model was applied to simulate all measured conditions. SRM decreased significantly in conditions A) and B) when the reference ITD was increased: In condition A) from 8.8 dB SNR on average at 0 ms reference ITD to 4.6 dB at 7 ms, in condition B) from 5.5 dB to 1.1 dB. In condition C) no significant effect was found. These results were accurately predicted by the applied EC-model. The outcomes show that interaural processing latency differences should be considered in asymmetric treatment of hearing loss.

## Introduction

Both sound localization and spatial release from masking (SRM) rely on precise timing of the signals reaching both ears and on symmetrical frequency-place mapping. However, listeners with hearing loss who use a hearing device only in one ear or who are provided with different devices in both ears can suffer from vast asymmetric hearing perceptions. In this study, we focus mainly on temporal asymmetries. For example, hearing instruments like hearing aids (HAs) and cochlear implants (CIs) can have large processing delays in the order of several milliseconds, which depend on their exact implementation. Timing differs due to processing delays but also due to physiologic latencies of acoustic hearing introduced by the ear canal, middle ear and mainly the cochlear delays in the inner ear (Ruggero & Temchin, 2007), which are not present when the auditory nerve is directly excited by electric current in the case of CI stimulation. The impact of this temporal alteration on subjective disturbance or sound quality has been studied extensively for provision with HAs (Agnew & Thornton, 2000; Bramsløw, 2010; Groth & Søndergaard, 2004; Stone & Moore, 2003; Stone, Moore, Meisenbacher, & Derleth, 2008). However, most of the studies were looking at maximal preferable delays and did not take the possibility of asymmetric timing between both ears into account. In treatment of asymmetric hearing loss, the ears often must be provided with different technical devices. Examples for this are unilateral hearing loss which requires provision with a HA on only one ear, treatment of single-sided deafness with a CI, or bimodal provision with a CI in one ear and a contralateral HA. Besides interaural differences in signal processing and sound information (e.g. fine structure and spectral information), a considerable interaural difference typically is a static temporal mismatch of the ear signals. For bimodal listeners provided with a MED-EL CI and a contralateral HA, an average interaural temporal difference of 7 ms in the neural representation of incoming signals at the level of the brainstem was reported (Zirn, Arndt, Aschendorff, & Wesarg, 2015). Furthermore, interaural differences in latency in the millisecond range have recently been found in some hearables (Denk, Schepker, Doclo, & Kollmeier, 2020). This interaural temporal mismatch severely impairs the ability of bimodal listeners to localize sounds in the frontal horizontal hemisphere. Equalizing the device delay mismatch by delaying the CI stimulation according to the measured HA processing delay resulted in highly significant improvements in the root-mean-square error and in the bias of localization judgements (Angermeier, Hemmert, & Zirn, 2021; Zirn, Angermeier, Arndt, Aschendorff, & Wesarg, 2019). The static interaural temporal mismatch will further be referred to as reference interaural time difference (ITD), i.e., the ITD at 0° azimuth, which is close to 0 µs when no temporal interaural asymmetry is present. What has not yet been systematically studied is the influence of large reference ITDs on speech understanding in noise, especially SRM. For SRM, processing of ITDs and interaural level differences (ILDs) contribute to unmasking when speech and noise are spatially separated (Lavandier & Best, 2020).

In listeners provided unilaterally with a HA or two different HAs with different processing latencies, ITDs are conveyed by the HAs and can be used for SRM alongside with ILDs. It has to be noted that these ITDs can be distorted in case of an open fitting, due to direct sound through the open earpiece overlapping with the delayed amplified signal (Denk, Ewert, & Kollmeier, 2019). On the other hand, in HA users where the residual hearing in the lower frequencies is sufficient and no amplification is needed by the HA, processing latencies will not affect ITD processing. Bimodal listeners, however have very limited access to ITDs (Francart & McDermott, 2013; Veugen, Chalupper, Snik, Opstal, & Mens, 2016). Therefore, bimodal users rely mostly on ILDs for SRM. Dieudonné & Francart (2020) have reported that SRM in bimodal listeners is mostly driven by monaural head shadow effects, i.e., one ear having access to a better signal-to-noise ratio when noise and speech are spatially separated. This effect should theoretically not be affected by an additional reference ITD.

Differences in the accessibility of cues in the presence of a reference ITD makes it interesting to examine the cues facilitating SRM separately. This study aimed to investigate if and how different reference ITDs affect SRM. To get a more complete picture of how the different binaural mechanisms underlying this process are affected by these asymmetries, tests were conducted with cues presented separately: A) ITDs and ILDs, B) ITDs only or C) ILDs only. SRM without a reference ITD but with respect to the available cues has been measured in prior studies. Bronkhorst & Plomp (1988) showed a substantial SRM for speech and laterally displaced noise of 7.8 dB when only ILDs were available, a 5 dB SRM difference when ITDs were available and 10.1 dB when both cues could be utilized together. Further studies investigated the role of different cues exploited for SRM with symmetrically placed maskers to reduce better-ear listening with symmetrically placed speech maskers (Ellinger, Jakien, & Gallun, 2017; Glyde, Buchholz, Dillon, Cameron, & Hickson, 2013; Kidd, Mason, Best, & Marrone, 2010). These studies found that ITDs and ILDs alone were sufficient to elicit SRM.

Another objective of this study was to determine how well the effect of a reference ITD on SRM can be predicted by an existing phenomenological computational spatial unmasking model. Computational binaural models have already been successfully applied in the past to accurately predict SRM in hearing-impaired listeners (Beutelmann, Brand, & Kollmeier, 2010; Vicente, Buchholz, & Lavandier, 2021; Williges, Dietz, Hohmann, & Jürgens, 2015; Zedan, Williges, & Jürgens, 2018). If a model can replicate the experimental results in normal hearing listeners, it might also be a valuable tool to predict outcomes in unilateral HA users or even in CI users with single-sided deafness (SSD) and bimodal listeners.

## Methods

### Test Environment and Stimuli

**Figure 1.**
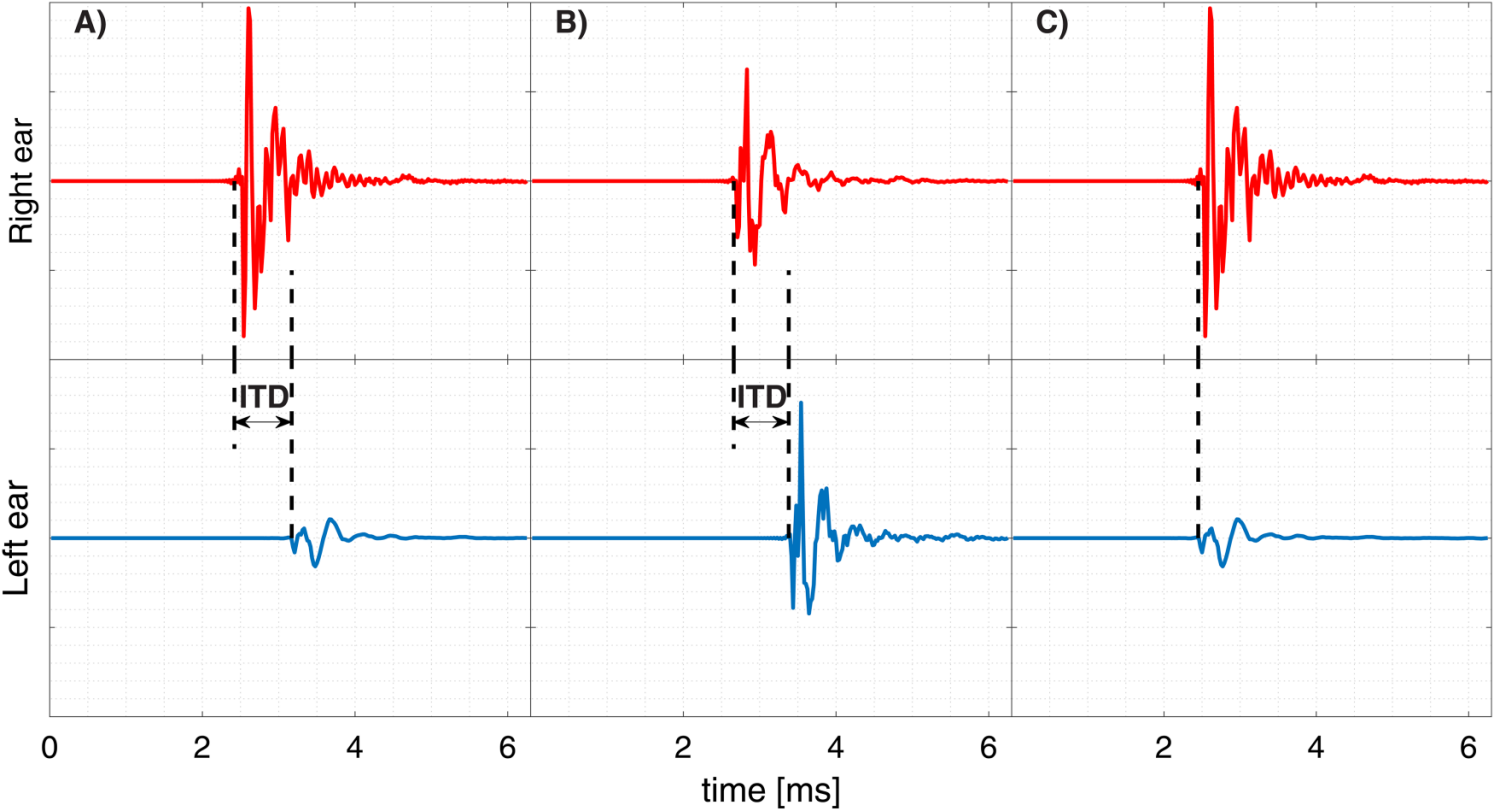
Head-related impulse responses at 90° azimuth for the different cue conditions (A: ITD and ILD; B: only ITD; C: only ILD).

All tests were conducted in an audiometric booth. Speech reception thresholds (SRT) were measured using the Oldenburg Sentence Test (OlSa), a German matrix sentence test (Wagener, Brand, & Kollmeier, 1999). To measure SRM, subjects performed speech tests with noise and speech coming from the front (S_0_N_0_) and speech coming from the front and noise coming from 90° azimuth (S_0_N_90_). Olnoise was used as a masker which is composed by overlaying the jittered speech material of the OlSa sentences 30 times resulting in a broadband noise with the same long-term average speech spectrum as the speech material. Speech material and noise were presented via Sennheiser HD 280 Pro headphones with a fixed noise level of 65 dB SPL and the speech level adaptively varied according to the subjects’ answers. Subjects used a tablet computer displaying the word material of the OlSa to input their answers. The answers were sent to a computer outside the audiometric booth via Bluetooth and processed on a computer using MATLAB (The MathWorks Inc., Natick, MA, USA). In-ear head related impulse responses (HRIRs) were used to allow for spatial separation of speech and noise (Kayser et al., 2009). A reference ITD was introduced by delaying the signals on the right ear channel by 0, 1.75, 3.5, 5.25 and 7 ms. To further investigate the effect of the reference ITD on different cues used for spatial unmasking, the HRIRs were manipulated according to Kulkarni et al. (1999) and Ellinger et al. (2017) to only offer either A) ITDs and ILDs, or B) ITDs only, or C) ILDs only. In the A) condition the HRIRs for 0° and 90° azimuth were not manipulated. In the B) condition for 90° azimuth the right ear channel of the HRIR at 0° azimuth was delayed by the delay found in the HRIR at 90° azimuth, thus not offering any ILDs and purely ITDs. In the C) condition the right ear channel of the HRIR at 90° azimuth was shifted by the delay such that ILDs were present, while removing ITDs. A graphical representation can be found in Figure 1.

### Experimental Procedure

Prior to the speech tests pure tone audiometry was performed at the frequencies of 0.5, 1, 2 and 4 kHz to ensure normal hearing in the participants according to WHO standards (Olusanya, Davis, & Hoffman, 2019). For each reference ITD, each condition (A, B, C) and each spatial scenario, two OlSa lists of 20 sentences were measured. This resulted in a total of 60 measured OlSa test lists per participant. Each combination of interaural cue and reference ITD was tested in blocks of 4 lists containing two lists in each condition, S_0_N_0_ and S_0_N_90_, with both lists being averaged for the calculation of the SRM. The presentation was randomized within and between these blocks. To avoid fatigue effects on the results the testing was done in up to 3 sessions lasting approximately 3 h with breaks after each block or if the subjects desired a break. Two training lists of 20 OlSa sentences were applied at the beginning of each session.

### Subjects

Ten normal hearing subjects (mean age: 25.6 ± 6; min: 21; max: 42; 2 female; 8 male) participated in this study. Their hearing threshold at 0.5, 1, 2, and 4 kHz did not exceed 20 dB HL with mean thresholds of 8.4 ± 1 dB HL for the right ear and 7.5 ± 0.7 dB HL for the left ear. All subjects provided written informed consent prior to their participation. All testing was conducted in accordance with the Code of Ethics of the World Medical Association (Declaration of Helsinki) for experiments involving humans and approved by the Technical University of Munich ethics committee (340/19).

### Simulations

#### Model Structure

For each condition, SRTs were simulated using a blind equalization-cancellation (EC) model (Hauth, Berning, Kollmeier, & Brand, 2020) implemented in the auditory modeling toolbox (Majdak, Hollomey, & Baumgartner, 2021). The model uses the mixed speech and noise signals of the right and left ear channels as inputs and splits them into 30 ERB spaced frequency bands between 150 Hz and 8500 Hz using a gammatone filterbank (Hohmann, 2002). The frequency bands up to 1500 Hz are then fed into an EC mechanism (Durlach, 1963) to model binaural unmasking. The cancellation is performed either by subtracting (level-minimization) or adding (level-maximization) the equalized left and right ear channel to account for negative as well as positive SNRs. After this step a blind decision stage based on a modulation analysis by Santos, Senoussaoui, & Falk (2014) is used to select whether the level-minimization or the level-maximization yields the better SNR. The selected path is then used for further processing. The frequency bands above 1500 Hz are only used for a better-ear processing with a SRMR selector to determine which ear channel has the better SNR. Both paths (EC and better ear) are then combined via a gammatone synthesis filterbank to be further processed by the back end of the model. As a back end the speech intelligibility index (SII; ANSI S3.5-1997) was used. For more details concerning the model structure, see Hauth et al. (2020).

#### Modeling Parameters and SRT Calculations

Each measured combination of noise azimuth, utilized cue and reference ITD was modeled. To further extend the modeling predictions all modeling was additionally done at 10 ms reference ITD. For each condition the mixed speech and noise were used as an input to the model at 21 SNRs between 0 and  − 20 dB SNR at 1 dB steps. For each SNR, ten sentences of the OlSa were presented to make sure every word of the OlSa speech material appeared once. Further, 10 Monte-Carlo simulations were run per sentence to account for the random jitter in the EC process of the model. This process resulted in 2100 simulations per combination of noise azimuth, cue, and reference ITD, with an overall number of 69300 simulations for all combinations. The SRT was calculated as described by Hauth et al. (2020) by using the intersection of the mean SII over all Monte-Carlo simulations and sentences and the mean experimental SRT measured at S_0_N_0_ without a reference ITD and with both spatial cues available. The resulting SII was further used as a reference SII for all other conditions modeled.

To investigate the influence of more realistic environments, especially the influence of reverberation, the model was also applied with an in-ear HRIR set recorded in a cafeteria within the same HRIR database by Kayser et al. (2009). In this setup the head and torso simulator (HATS) used to record the HRIRs was seated at a table with the target speaker at 0° azimuth opposite to it. The distance between HATS and target speaker was 102 cm. The target speaker faced the HATS. The spatially separated speaker was located at 90° azimuth to the HATS at a distance of 52 cm. In the original HRIR dataset this speaker is at  − 90° but the room was flipped by switching the right-ear and left-ear channel of the HRIRs. The spatially separated speaker was oriented towards the frontal speaker. For the given cafeteria setting the reverberation time T_60_ was 1250 ms. For this setting reference ITDs were to the same as the ones applied in the anechoic condition. For SRT extraction the experimentally determined reference SII from anechoic measurements was used.

### Statistical Analysis

Non-parametric Friedman tests with an alpha level of 0.05 were used to test for differences between different reference ITDs in the measured SRTs and SRM results for all conditions. In the case of significant outcomes post-hoc pairwise testing was applied via Wilcoxon signed-rank tests with a Bonferroni-Holm correction for multiple comparisons. To compare the measured data with the modeling results linear regression was performed. We performed all statistical testing in MATLAB.

## Results

### Experimental Results

**Figure 2.**
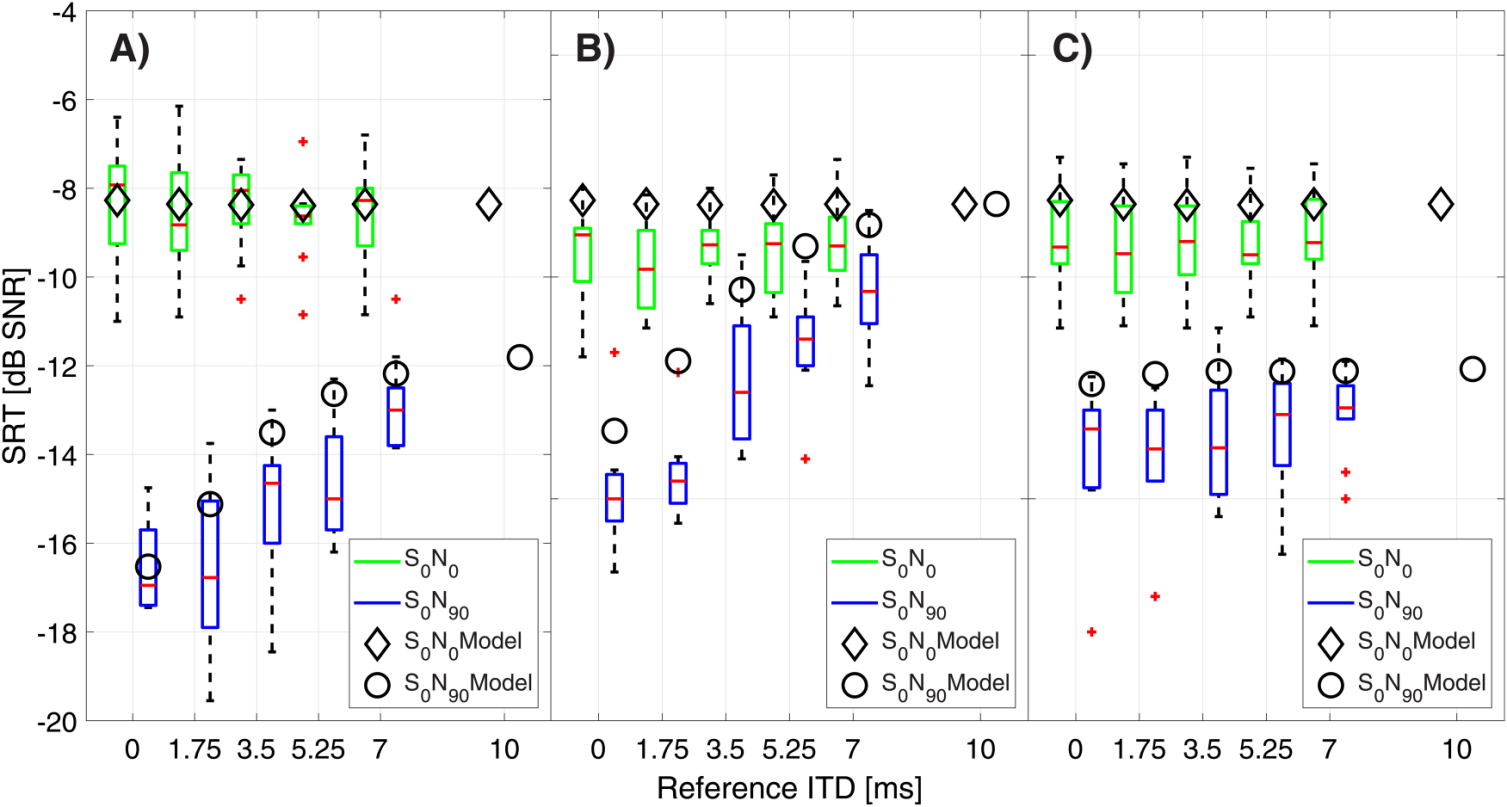
Measured speech reception thresholds (SRT) in ten normal hearing subjects for reference ITD of 0, 1.75, 3.5, 5.25 and 7 ms as boxplots (red line: median; box: 1^st^–3^rd^ quartile; whiskers: minimum and maximum without outliers; outliers in red) for either collocated speech and noise at 0° azimuth (green boxes) or speech at 0° and noise at 90° azimuth (blue boxes). Modeled SRTs for collocated speech and noise are denoted by diamonds, and for spatially separated speech and noise by circles. Modeled SRTs depicted up to 10 ms reference ITD. A) ITDs & ILDs condition B) ITDs only condition C) ILDs only condition.

Figure 2 shows the measured SRTs as boxplots for the respective cue available to the listeners. For each reference ITD the green boxplots correspond to the S_0_N_0_ condition, and the blue boxplots correspond to the S_0_N_90_ condition. In the S_0_N_0_ condition Friedman tests revealed no significant difference for rising reference ITD for the condition A), with both cues available (χ2(4) = 5.56 p = 0.2), B) (χ2(4) = 7.98 p = 0.09), and C) (χ2(4) = 7.56 p = 0.1). In the spatially separated condition significant differences for differing reference ITD could be seen in condition A) (χ2(4) = 34.71 p < 0.001) and in condition B) (χ2(4) = 37.01 p <0.001). If only ILDs were present (condition C)), a rising reference ITD did not lead to a significant change in the measured SRTs (χ2(4) = 7.67 p = 0.1). Test-retest reproducibility was calculated as mean absolute difference between the two measured testlists per condition, reference ITD and spatial configuration revealing 0.7 ± 0.2 dB in condition A) 0.7 ± 0.1 dB in condition B) and 0.9 ± 0.2 dB in condition C). No significant differences were found between the conditions.

**Figure 3.**
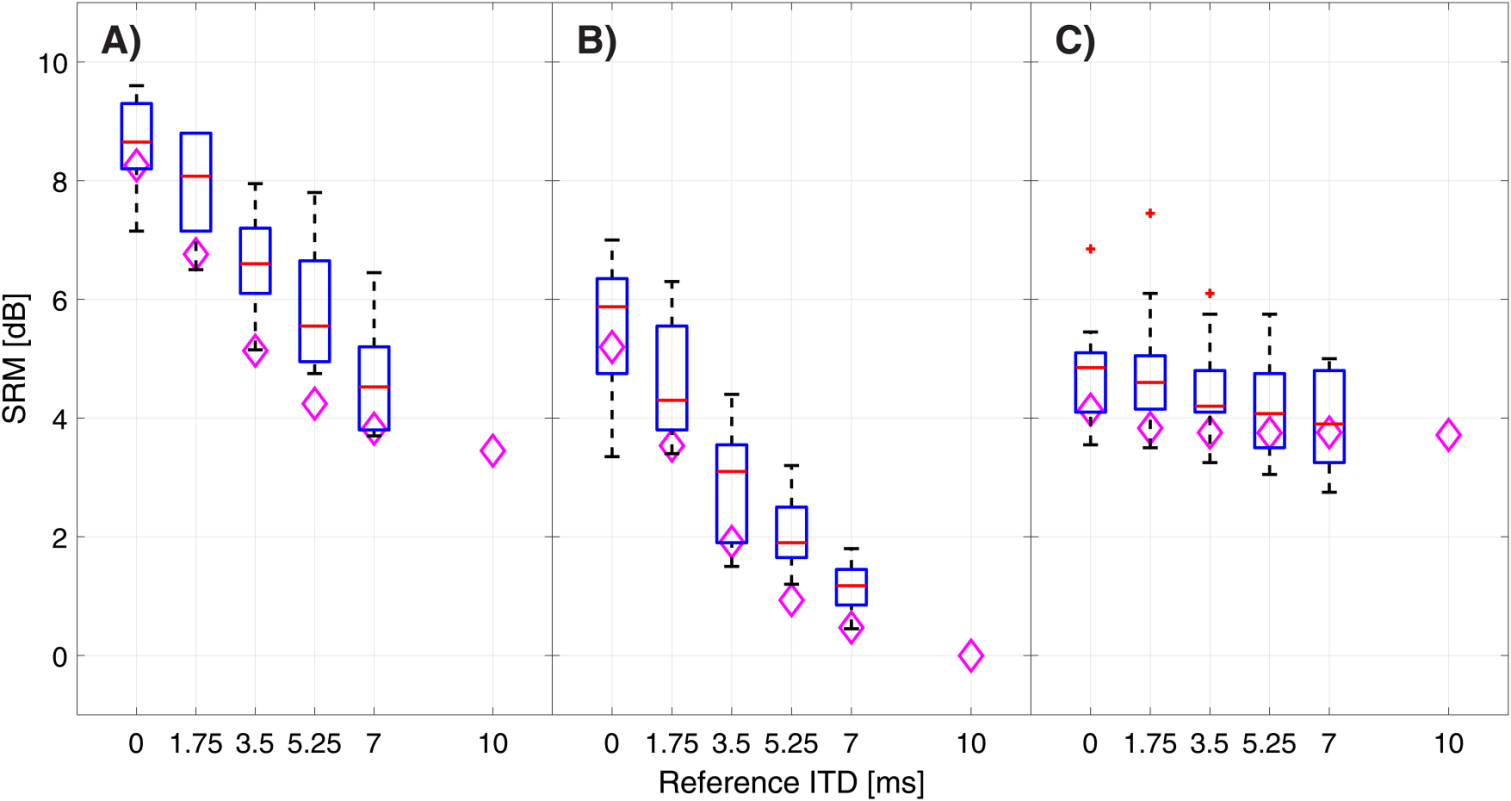
Measured spatial release from masking (SRM) in ten normal hearing subjects for reference ITD of 0, 1.75, 3.5, 5.25 and 7 ms as boxplots (red line: median; box: 1^st^–3^rd^ quartile; whiskers: minimum and maximum without outliers; outliers in red). Modeled SRM denoted by diamonds. Modeled SRM depicted up to 10 ms reference ITD. A) ITDs & ILDs condition B) ITDs only condition C) ILDs only condition.

Figure 3 shows the calculated SRM for each of the cue conditions. In condition A), the mean SRM at 0 ms reference ITD was 8.82 dB with a standard deviation of 1.12 dB. With a rising reference ITD the SRM decreased to 4.63 ± 0.89 dB at 7 ms reference ITD. Friedman tests revealed a significant influence of reference ITD in this condition (χ2(4) = 37.61 p < 0.01). For condition B), the SRM at 0 ms was 5.48 ± 1.21 dB and decreased to 1.12 ± 0.41 dB at a reference ITD of 7 ms. This effect also proved highly significant (χ2(4) = 35.04 p < 0.01). In condition C), the SRM at 0 ms reference ITD was 4.81 ± 0.97 dB and slightly decreased at 7 ms reference ITD to 3.94 ± 0.81 dB. The SRM in this condition was also significantly affected by the rising temporal asymmetry between both ears (χ2(4) = 10.64 p = 0.031). Pairwise comparisons showed significant differences between all measured SRM values in condition A) (p < 0.05). In condition B) only the differences in SRM at 0 ms versus 1.75 ms (p = 0.09) and at 3.5 ms versus 5.25 ms reference ITD (p = 0.09) did not prove significant. In condition C) none of the tested differences proved to be significant. We hypothesize this to be due to better-ear listening dominating the SRM, which is not influenced by a rising reference ITD, given its monaural nature. At a reference ITD of 7 ms, there was almost no more SRM due to ITDs in condition A) and the SRM measured approached the SRM caused by ILDs seen in condition C) which was not influenced by the reference ITD.

### Modeling Results

Figure 2 shows the modeled SRTs for S_0_N_0_ as diamonds and for S_0_N_90_ plotted as circles. The model's behavior matched the experimental results qualitatively. For S_0_N_0_ the coefficients of determination (R^2^) and root-mean-square errors (RMSE) are reported in Table 1. The low values for R^2^ in the conditions B) and C) are not surprising given the shallow slope within the datasets. For the S_0_N_90_ condition the coefficient of determination were generally much higher as Table 1 shows. The additional modeled SRTs at a reference ITD of 10 ms show further deterioration in the S_0_N_90_ condition in conditions A) and B) but not in condition C).

**Table 1.** Coefficients of Determination (R^2^) and Root-Mean-Square Errors (RMSE) for the Linear Regression Between Modeled and Measured Results. A) ITDs & ILDs Condition B) ITDs Only Condition C) ILDs Only Condition.

	A		B		C		
	R^2^	RMSE [dB]	R^2^	RMSE [dB]	R^2^	RMSE [dB]
**S_0_N_0_**	0.6	0.03	0.02	0.05	0.07	0.05
**S_0_N_90_**	0.92	0.6	0.93	0.5	0.3	0.1
**SRM**	0.95	0.5	0.97	0.4	0.38	0.2

Figure 3 depicts a comparison between the modeled SRM and the measured SRM. Linear regression was performed to determine the accuracy of the model. The corresponding coefficients of determination and RMSEs for the comparison between modeled and measured SRM can be found in Table 1. The additionally modeled SRM for a reference ITD of 10 ms show that a reference ITD of 10 ms eliminates SRM based on ITD as seen in condition B).

**Figure 4.**
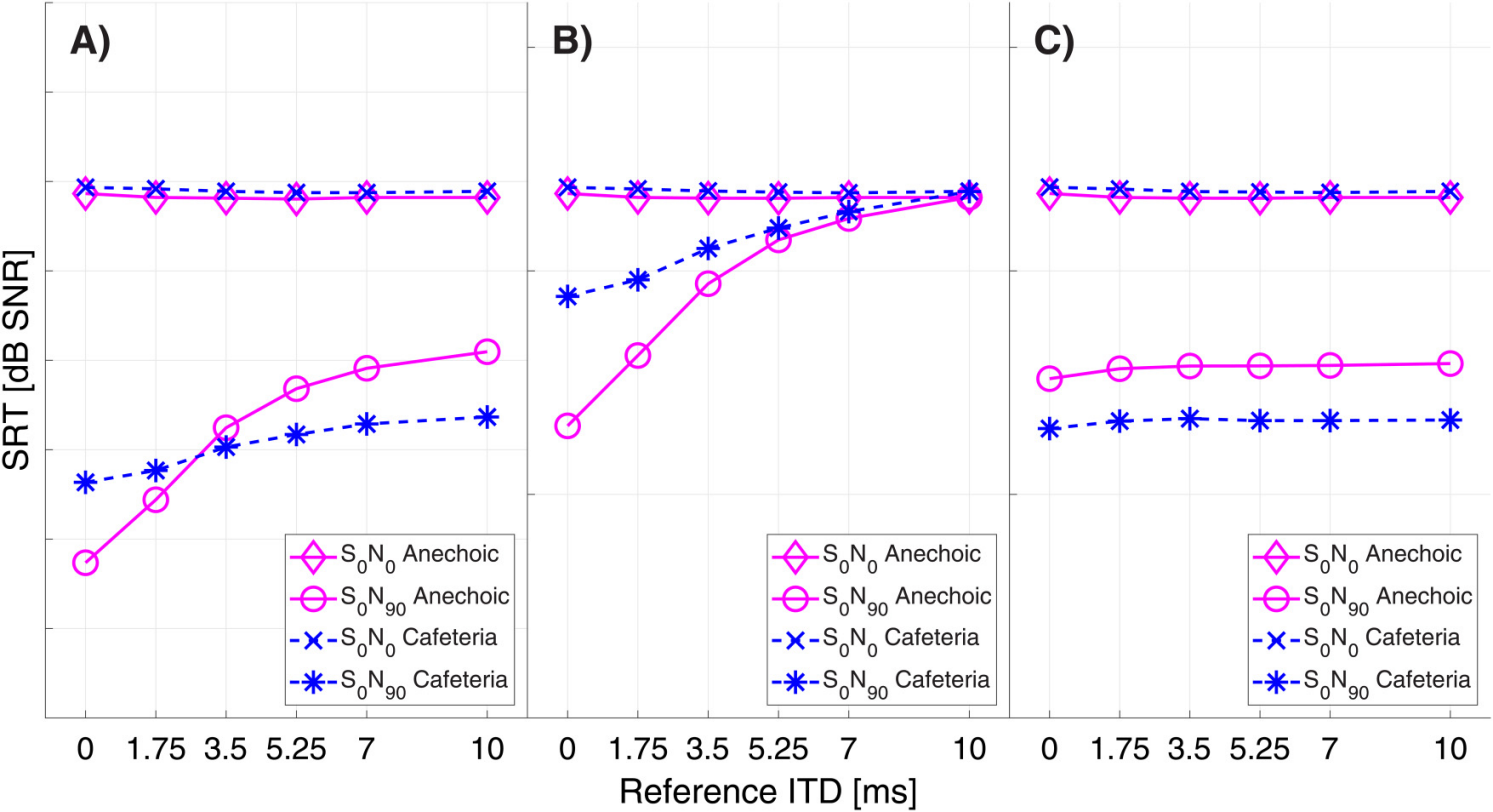
Modeled speech reception thresholds (SRT) for reference ITDs of 0, 1.75, 3.5, 5.25, 7 & 10 ms for spatially collocated and spatially separated target and masker in an anechoic setting (magenta) and a reverberant cafeteria environment (blue). A) ITDs & ILDs condition B) ITDs only condition C) ILDs only condition.

In Figure 4 the modeled SRTs for S_0_N_0_ and S_0_N_90_ condition are depicted for the anechoic condition in magenta and the reverberant (cafeteria) condition in blue. For S_0_N_0_ condition the difference between anechoic condition and cafeteria condition was quite low with a mean absolute difference of 0.15 dB for condition A), 0.14 dB for condition B) and 0.14 dB for condition C). In the S_0_N_90_ condition, the mean absolute difference between the anechoic condition and the cafeteria condition was 1.1 dB for condition A), 0.9 dB for condition B) and 1.2 dB in condition C).

**Figure 5.**
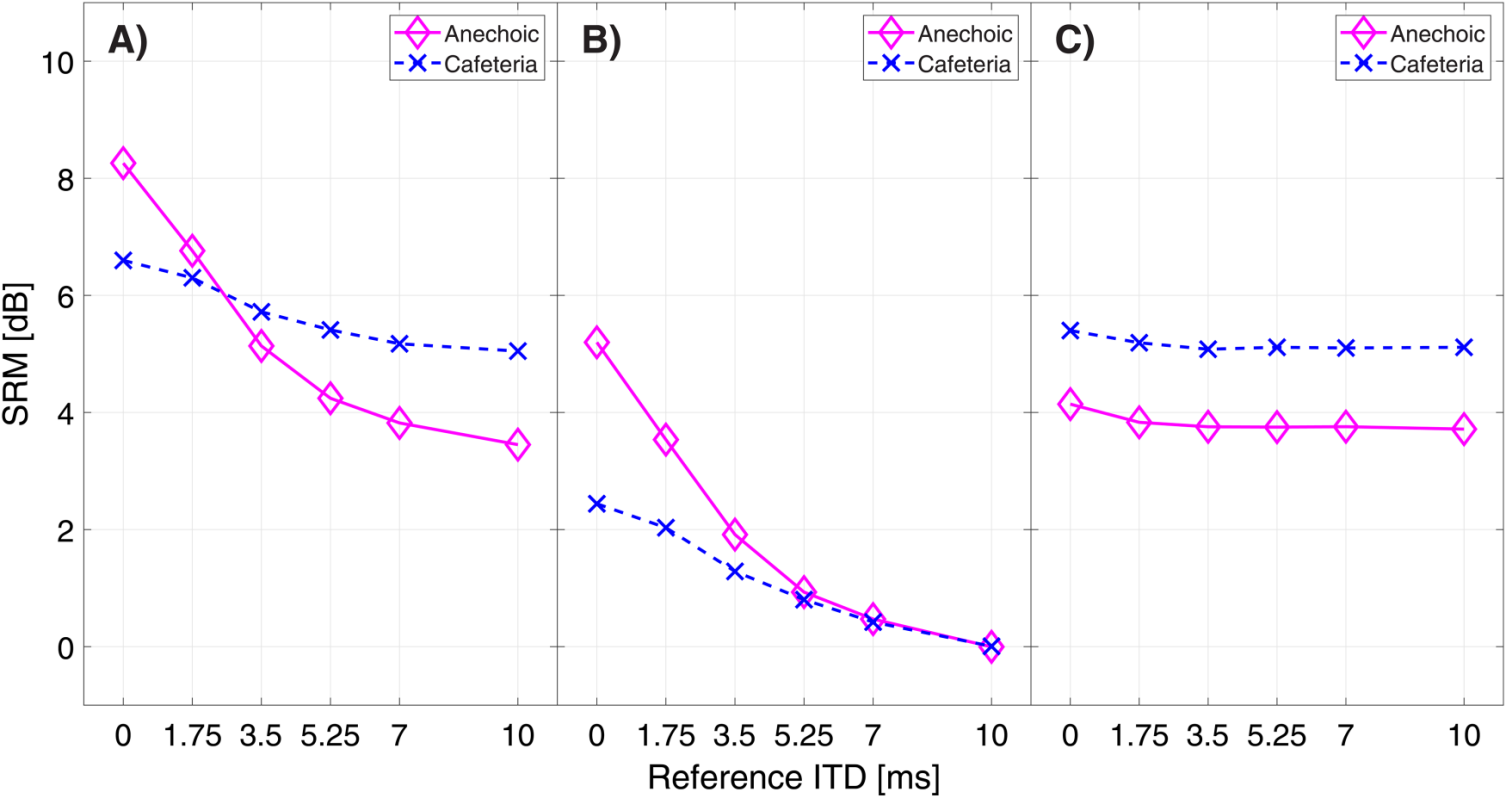
Modeled spatial release from masking (SRM) for reference ITDs of 0, 1.75, 3.5, 5.25, 7 & 10 ms in an anechoic setting (magenta) and a reverberant cafeteria environment (blue). A) ITDs & ILDs condition B) ITDs only condition C) ILDs only condition.

Figure 5 displays the modeled SRM for the anechoic environment in magenta and the cafeteria environment in blue. For condition A) the initial SRM at 0 ms reference ITD is 8.3 dB in the anechoic environment and 6.6 dB in the cafeteria environment. At a reference ITD of 10 ms the model predicted SRM of 3.5 dB for the anechoic environment and 5.1 dB in the cafeteria environment, showing a lower influence of reference ITD on SRM in the reverberant environment. When only ITDs were present in the signal (condition B)), SRM at 0 ms reference ITD is at 5.2 dB in the anechoic environment and at 2.4 dB in the cafeteria environment. At 10 ms reference ITD both SRM in the anechoic environment and in the cafeteria were at 0 dB in this condition. With only ILDs present in the signals SRM for the anechoic environment was 4.1 dB at 0 ms reference ITD and slightly deteriorated to 3.7 dB at 10 ms reference ITD. In the cafeteria environment SRM by ILD was at 5.4 dB for a reference ITD of 0 ms and at 5.1 dB at 10 ms reference ITD.

## Discussion

In this study, the influence of reference ITDs of several milliseconds on SRM was investigated. Unilateral delays in this range were found in bimodal HA/CI users (Zirn et al., 2015). To deepen the understanding of the effect of a reference ITD, all measurements were conducted with the different binaural cues that enable SRM separately. Further we investigated whether the measured effects can be reproduced by an EC-model for binaural unmasking. The results showed that an increasing reference ITD led to a significant decrease in SRM, which affected unmasking by ITDs more drastically than unmasking though ILDs. When both cues were available to the subjects, unmasking due to ITDs and ILDs were roughly additive. Furthermore, the measured results were accurately predicted by an EC-model. When a reverberant environment was used with the model the decrease in SRM by reference ITDs was less drastic than in an anechoic condition.

In contrast to previous studies investigating SRM with isolated cues, the reported results showed that with a reference ITD of 0 ms, SRM achieved when ITDs were used to separate target and masker was bigger than the SRM achieved when only ILDs were present in the signal. These differences probably originated from different masker types. Glyde et al. (2013) applied symmetrically placed speech maskers to measure SRM, whereas Bronkhorst & Plomp (1988) used a single source of masking noise with a speech envelope. Both maskers allow the use of dynamic head shadow in the quiet parts of the masker, allowing better speech understanding when only ILDs are present compared to ITDs alone. These methodological differences would require further experiments to allow for sufficient comparisons between our data and the data reported in the literature.

The presented results reveal a limiting factor to SRM in listeners with asymmetric treatment of hearing loss, which has not yet been reported. A reference ITD in the range of a few milliseconds has already significant detrimental effects on the unmasking of speech when ITDs and ILDs are conveyed sufficiently by the hearing devices. In the ITDs only condition (B), a reference ITD of 7 ms almost eliminated SRM. In the ILDs only condition (C) a reference ITD did not influence SRM significantly, processing in the auditory system in this condition seems more robust against a reference ITD. However, with binaural measurements it is only possible to differentiate between SRM based on binaural mechanisms and access to one ear having a better signal to noise ratio, which is a monaural mechanism to some extent. To disentangle these mechanisms, monaural measurements could be utilized to measure the effect of head shadow as proposed by Dieudonné & Francart (2019). The present findings also suggest that, while SRM based on ITDs diminishes for a reference ITD of 7 ms, considerable SRM remains due to better-ear listening. In bimodal listeners provided with a CI and a contralateral HA, the device delay mismatch between HA and CI is expected to have no big impact on SRM, since only ILDs and not ITDs are sufficiently conveyed by the CI (Francart & McDermott, 2013; Veugen et al., 2016). Consequently it has been shown that SRM in bimodal listeners is driven by head shadow (Williges et al., 2019). However, as soon as more sophisticated CI coding strategies code ITDs with higher fidelity in the future, the impact of a reference ITD on SRM needs to be considered as a significant limiting factor, especially since significant deterioration of SRM can already be seen at a reference ITD of 1.75 ms. This leads to the hypothesis that, when both cues are available for listeners, a matching accuracy of processing latencies in their respective devices below this reference ITD of 1.75 ms should be considered favorable.

The applied model was able to predict the experimental data with sufficient precision. This is probably due to the combination of an EC-processing and a monaural SRMR processing, where the monaural part still offers substantial SRM in the presence of ILDs when the EC-processing fails to deliver release from masking. The detrimental effect of a reference ITD on the EC-processing can be easily explained by the processing errors included in this model, that were originally proposed by Vom Hövel (1984). These processing errors for the estimation of ITDs are dependent on the actual ITDs in the signal. Thus, as the reference ITD increases, the processing errors of the ITD equalization within the model increase, making the equalization less accurate. The level equalization, however, is not affected by the reference ITD since mathematically, the ILD processing errors are dependent on the ILDs that are found within the signal. This allows the model to accurately describe the effects that can be measured experimentally. The low value for R^2^ in the ILDs only condition can be explained due to the measured data being explained better by its own mean than by the model, which is not surprising as to the SRM was only minimally affected by an increasing reference ITD. However, the low RMSE in this condition still demonstrated a good accuracy of the model. Overall, the model can predict the measured SRM in the presence of a reference ITD of several milliseconds with high precision.

In future studies the model by Hauth et al. (2020) could possibly be used to model performance in listeners with asymmetric hearing loss in which a reference ITD greater than 0 ms occurs due to the asymmetries in treatment. Williges et al. (2015) and Zedan et al. (2018) both used EC-based models to accurately predict SRM in simulated bimodal listeners, given some additional preprocessing of the signal to simulate the CI and the HA. These results in combination with the high accuracy the model provides in normal hearing listeners supports the hypothesis that modeling the effects of a reference ITD in hearing impaired listeners with EC-based models is possible and that models are a valuable tool for the prediction of treatment outcomes.

When comparing the modeling results in an anechoic environment to a more realistic reverberant scenario the influence of a rising reference ITD is much lower. This is due to the SRM based on ITD being vastly diminished in a reverberant environment. The influence of reverberation on ITD processing has been shown for fine structure ITD (Devore & Delgutte, 2010) and for envelope ITD (Monaghan, Krumbholz, & Seeber, 2013). In the ILD only condition SRM was better in the reverberant environment than in the anechoic environment irrespective of the reference ITD, suggesting that ILDs play a more important role in spatial unmasking in reverberation. However even in the reverberant environment an overall SRM decay of 1.6 dB between 0 and 10 ms reference ITD can be observed when both ILD and ITD are present in the signal. To further verify whether these modeling results can be considered realistic, more experimentation in the same reverberant conditions with normal hearing listeners should be undertaken in the future.

## Conclusion

In normal hearing listeners, a reference ITD has a significant detrimental influence on SRM. Our results show that this reference ITD mainly impairs the effect of ITDs in the signal on SRM. When only ILDs were presented, a reference ITD of a few milliseconds showed no significant effect on SRM. These results may be particularly relevant for bimodal listeners with different devices in each ear. The measured results can be accurately predicted by an EC-based model from Hauth et al. (2020) for binaural unmasking of speech. In a reverberant environment, modeling showed a smaller but still detrimental effect of reference ITD on SRM, mainly due to the small proportion of SRM based on ITD in reverberation. These results however should be verified experimentally.

## References

[bibr1-23312165221094202] Agnew J. Thornton J. M. (2000). Just noticeable and objectionable group delays in digital hearing aids. Journal of the American Academy of Audiology, 11(6), 330–336. PMID: 10858005. 10858005

[bibr2-23312165221094202] Angermeier J. Hemmert W. Zirn S . (2021). Sound localization bias and error in bimodal listeners improve instantaneously when the device delay mismatch is reduced. Trends in Hearing, 25, 23312165211016164. 10.1177/23312165211016165 PMC8182625 34057366

[bibr3-23312165221094202] ANSI S3.5-1997 (1997). Methods for the Calculation of the Speech Intelligibility Index.

[bibr4-23312165221094202] Beutelmann R. Brand T. Kollmeier B . (2010). Revision, extension, and evaluation of a binaural speech intelligibility model. The Journal of the Acoustical Society of America, 127(4), 2479–2497. 10.1121/1.3295575 20370031

[bibr5-23312165221094202] Bramsløw L . (2010). Preferred signal path delay and high-pass cut-off in open fittings. International Journal of Audiology, 49(9), 634–644. 10.3109/14992021003753482 20602601

[bibr6-23312165221094202] Bronkhorst A. W. Plomp R . (1988). The effect of head-induced interaural time and level differences on speech intelligibility in noise. The Journal of the Acoustical Society of America, 83(4), 1508–1516. 10.1121/1.395906 3372866

[bibr7-23312165221094202] Denk F. Ewert S. D. Kollmeier B . (2019). On the limitations of sound localization with hearing devices. The Journal of the Acoustical Society of America, 146(3), 1732–1744. 10.1121/1.5126521 31590539

[bibr8-23312165221094202] Denk F. Schepker H. Doclo S. Kollmeier B . (2020). Acoustic transparency in hearables—technical evaluation. Journal of the Audio Engineering Society, 68(7/8), 508–521. 10.17743/jaes.2020.0042

[bibr9-23312165221094202] Devore S. Delgutte B . (2010). Effects of reverberation on the directional sensitivity of auditory neurons across the tonotopic axis: influences of interaural time and level differences. The Journal of Neuroscience, 30(23), 7826–7837. 10.1523/JNEUROSCI.5517-09.2010 20534831 PMC2896784

[bibr10-23312165221094202] Dieudonné B. Francart T . (2019). Redundant information is sometimes more beneficial than spatial information to understand speech in noise. Ear and Hearing, 40(3), 545–554. 10.1097/AUD.0000000000000660 30299342

[bibr11-23312165221094202] Dieudonné B. Francart T . (2020). Speech understanding with bimodal stimulation is determined by monaural signal to noise ratios: No binaural cue processing involved. Ear & Hearing, 41(5), 1158–1171. 10.1097/AUD.0000000000000834 32833388

[bibr12-23312165221094202] Durlach N. I . (1963). Equalization and cancellation theory of binaural masking-level differences. The Journal of the Acoustical Society of America, 35(8), 1206–1218. 10.1121/1.1918675

[bibr13-23312165221094202] Ellinger R. L. Jakien K. M. Gallun F. J . (2017). The role of interaural differences on speech intelligibility in complex multi-talker environments. The Journal of the Acoustical Society of America, 141(2), EL170–EL176. 10.1121/1.4976113 PMC5392079 28253635

[bibr14-23312165221094202] Francart T. McDermott H. J . (2013). Psychophysics, fitting, and signal processing for combined hearing aid and cochlear implant stimulation. Ear & Hearing, 34(6), 685–700. 10.1097/AUD.0b013e31829d14cb 24165299

[bibr15-23312165221094202] Glyde H. Buchholz J. M. Dillon H. Cameron S. Hickson L . (2013). The importance of interaural time differences and level differences in spatial release from masking. The Journal of the Acoustical Society of America, 134(2), EL147–EL152. 10.1121/1.4812441 23927217

[bibr16-23312165221094202] Groth J. Søndergaard M. B . (2004). Disturbance caused by varying propagation delay in non-occluding hearing aid fittings. International Journal of Audiology, 43(10), 594–599. 10.1080/14992020400050076 15724524

[bibr17-23312165221094202] Hauth C. F. Berning S. C. Kollmeier B. Brand T . (2020). Modeling binaural unmasking of speech using a blind binaural processing stage. Trends in Hearing, 24, 1–16. 10.1177/2331216520975630 PMC7734536 33305690

[bibr18-23312165221094202] Hohmann V. (2002). Frequency analysis and synthesis using a gammatone filterbank. Acta Acustica United with Acustica, 88(3), 433–442.

[bibr19-23312165221094202] Kayser H. Ewert S. D. Anemüller J. Rohdenburg T. Hohmann V. Kollmeier B . (2009). Database of multichannel in-ear and behind-the-ear head-related and binaural room impulse responses. EURASIP Journal on Advances in Signal Processing, 2009(1), 1–10. 10.1155/2009/298605

[bibr20-23312165221094202] Kidd G. Mason C. R. Best V. Marrone N . (2010). Stimulus factors influencing spatial release from speech-on-speech masking. The Journal of the Acoustical Society of America, 128(4), 1965–1978. 10.1121/1.3478781 20968368 PMC2981113

[bibr21-23312165221094202] Kulkarni A. Isabelle S. K. Colburn H. S . (1999). Sensitivity of human subjects to head-related transfer-function phase spectra. The Journal of the Acoustical Society of America, 105(5), 2821–2840. 10.1121/1.426898 10335633

[bibr22-23312165221094202] Lavandier M. Best V. (2020). Modeling binaural speech understanding in Complex situations. In Blauert J. Braasch J. (Eds.), The technology of binaural understanding (pp. 547–578). Springer International Publishing. 10.1007/978-3-030-00386-9_19

[bibr23-23312165221094202] Majdak P. Hollomey C. Baumgartner R . (2021). AMT 1.0: The toolbox for reproducible research in auditory modeling. *Submitted to Acta Acustica*. Retrieved from https://amtoolbox.org/notes/MajdakHollomeyBaumgartner2021.pdf

[bibr24-23312165221094202] Monaghan J. J. M. Krumbholz K. Seeber B. U . (2013). Factors affecting the use of envelope interaural time differences in reverberation. The Journal of the Acoustical Society of America, 133(4), 2288–2300. 10.1121/1.4793270 23556596

[bibr25-23312165221094202] Olusanya B. O. Davis A. C. Hoffman H. J . (2019). Hearing loss grades and the international classification of functioning, disability and health. Bulletin of the World Health Organization, 97(10), 725–728. 10.2471/BLT.19.230367 31656340 PMC6796665

[bibr26-23312165221094202] Ruggero M. Temchin A . (2007). Similarity of traveling-wave delays in the hearing organs of humans and other tetrapods. Journal of the Association for Research in Otolaryngology : JARO, 8, 153–166. 10.1007/s10162-007-0081-z 17401604 PMC1868567

[bibr27-23312165221094202] Santos J. F. Senoussaoui M. Falk T. H . (2014). An improved non-intrusive intelligibility metric for noisy and reverberant speech. *2014 14th International Workshop on Acoustic Signal Enhancement (IWAENC)*, 55–59. 10.1109/IWAENC.2014.6953337

[bibr28-23312165221094202] Stone M. A. Moore B. C. J . (2003). Tolerable hearing aid delays. III. Effects on speech production and perception of across-frequency variation in delay. Ear and Hearing, 24(2), 175–183. 10.1097/01.AUD.0000058106.68049.9C 12677113

[bibr29-23312165221094202] Stone M. A. Moore B. C. J. Meisenbacher K. Derleth R. P . (2008). Tolerable hearing aid delays. V. Estimation of limits for open canal fittings. Ear and Hearing, 29(4), 601–617. 10.1097/AUD.0b013e3181734ef2 18469715

[bibr30-23312165221094202] Veugen L. C. E. Chalupper J. Snik A. F. M. van Opstal A. J. Mens L. H. M. (2016). Matching automatic gain control across devices in bimodal cochlear implant users. Ear and Hearing, 37(3), 260–270. 10.1097/AUD.0000000000000260 26656192

[bibr31-23312165221094202] Vicente T. Buchholz J. M. Lavandier M . (2021). Modelling binaural unmasking and the intelligibility of speech in noise and reverberation for normal-hearing and hearing-impaired listeners. The Journal of the Acoustical Society of America, 150(5), 3275–3287. 10.1121/10.0006736 34852607

[bibr32-23312165221094202] Vom Hövel H. (1984). Zur Bedeutung der Übertragungseigenschaften des Aussenohrs sowie des binauralen Hörsystems bei gestörter Sprachübertragung (p. III, 165, 27 S. : graph. Darst.). Aachen. Retrieved from https://publications.rwth-aachen.de/record/68790

[bibr33-23312165221094202] Wagener K. Brand T. Kollmeier B. (1999). Entwicklung und evaluation eines satztests in deutscher sprache III: evaluation des oldenburger satztests. Zeitschrift Für Audiologie, 38(3), 86–95.

[bibr34-23312165221094202] Williges B. Dietz M. Hohmann V. Jürgens T . (2015). Spatial release from masking in simulated cochlear implant users with and without access to low-frequency acoustic hearing. Trends in Hearing, 19, 1–14. 10.1177/2331216515616940 PMC4771029 26721918

[bibr35-23312165221094202] Williges B. Wesarg T. Jung L. Geven L. I. Radeloff A. Jürgens T . (2019). Spatial speech-in-noise performance in bimodal and single-sided deaf cochlear implant users. Trends in Hearing, 23, 233121651985831. 10.1177/2331216519858311 PMC6669847 31364496

[bibr36-23312165221094202] Zedan A. Williges B. Jürgens T . (2018). Modeling speech intelligibility of simulated bimodal and single-sided deaf cochlear implant users. Acta Acustica United with Acustica, 104(5), 918–921. 10.3813/AAA.919256

[bibr37-23312165221094202] Zirn S. Arndt S. Aschendorff A. Wesarg T . (2015). Interaural stimulation timing in single sided deaf cochlear implant users. Hearing Research, 328, 148–156. 10.1016/j.heares.2015.08.010 26302945

[bibr38-23312165221094202] Zirn Stefan Angermeier J. Arndt S. Aschendorff A. Wesarg T . (2019). Reducing the device delay mismatch can improve sound localization in bimodal cochlear implant/hearing-aid users. Trends in Hearing, 23, 233121651984387. 10.1177/2331216519843876 PMC6484236 31018790

